# Tuberculosis screening among persons with diabetes mellitus in Pune, India

**DOI:** 10.1186/s12879-017-2483-9

**Published:** 2017-06-02

**Authors:** Vidya Mave, Smita Nimkar, Haridas Prasad, Dileep Kadam, Sushant Meshram, Rahul Lokhande, Nikhil Gupte, Divyashri Jain, Amita Gupta, Jonathan E. Golub

**Affiliations:** 1 Byramjee-Jeejeebhoy Government Medical College-Johns Hopkins University Clinical Research Site, 1st Floor, Pathology Museum, Jai Prakash Narayan Road, Pune, Maharashtra 411001 India; 2 Johns Hopkins University School of Medicine, Baltimore, MD USA; 3 Byramjee-Jeejeebhoy Government Medical College, Pune, India

**Keywords:** Tuberculosis, Diabetes mellitus, Risk factors, Screening, India

## Abstract

**Background:**

Diabetes mellitus (DM) increases tuberculosis (TB) risk, and there is increasing concern over the public health implications of the convergence of these two epidemics. Screening for TB among people with DM is now recommended in India.

**Methods:**

People with DM seeking care at a large public sector tertiary care hospital clinic in Pune, India, were screened for TB from June 2015 to May 2016. All consenting people with DM were screened for TB at each clinic visit using a five-item, WHO-recommended questionnaire and those with TB symptoms and/or risk factors were tested for active TB using sputum smear microscopty, Xpert® MTB/RIF and TB culture. Categorical data and continuous variables were summarized using descriptive statistics. The *x* ^2^ test or Wilcoxon rank-sum test was used to ascertain significant associations between categorical and continuous variables, respectively.

**Results:**

Among 630 adults approached for screening, median age was 60 (interquartile range (IQR), 57–64) years and 350 (56%) were females. Median hemoglobin A1c (HbA1c) was 8.7% (IQR, 6.7–9.9) and 444 (70.5%) were poorly controlled DM (HbA1c > 7). Forty-four (7%) had prior history of TB but the proportion with TB risk factors at screening was low (<5%). While 18% of participants reported any TB symptoms, none of these patients were diagnosed with culture confirmed TB.

**Conclusions:**

Our study failed to yield any active TB cases using a WHO-recommended questionnaire among people with DM. High TB risk populations among people with DM must be identified if TB screening is to be feasible in settings such as India where the DM epidemic continues to rise.

**Electronic supplementary material:**

The online version of this article (doi:10.1186/s12879-017-2483-9) contains supplementary material, which is available to authorized users.

## Background

Tuberculosis (TB) has recently become the leading cause of mortality related to an infectious agent, even surpassing HIV [1]. While significant gains in global TB control have been achieved*,* an estimated 10.4 million people developed TB disease and 1.8 million died from the disease in 2015. With increasing burden of non-communicable diseases in low and middle income countries, overlap of conditions, such as diabetes mellitus (DM) and TB has risen, posing additional challenges to disease control and management [2]. Because DM increases TB risk by at least 3-fold and DM burden is increasing, there have been grave concerns that the DM epidemic may slow the decline of global TB incidence and may hinder the goal of achieving the global milestones of a 50% reduction in TB incidence and 75% reduction in TB deaths by 2025 [3–5]. Both the Bali declaration and the World Health Organization (WHO) and the International Union Against Tuberculosis and Lung Disease (IUATLD) joint statement supported a collaborative framework that recommended bidirectional screening including active TB case finding among people with DM [6, 7].

In 2015, India alone was estimated to have a staggering 69 million people aged 20–79 years affected with DM [8]. Furthermore, with an estimated over two million new cases annually, India accounts for 18% of the world’s estimated TB burden [1]. Therefore, the public health implications of the convergence of these two epidemics in India is paramount, and screening for TB among people with DM may be an important strategy. While several studies have evaluated the burden of DM among TB patients [9–13], data on the burden of TB among people with DM in India are limited [14]. In a multicenter cross-sectional study that screened people with DM for TB using a standard TB symptom-screen approach, 600–950 cases per 100,000 were detected, however whether such a strategy will yield similar results in different regions in India is not known. We present a study that aimed to screen for prevalent TB and TB risk factors among people with DM in western India using WHO recommended TB screening questionnaire [1]. Our study further conducted sputum smear microscopy, gene Xpert and TB culture investigations among those with positive TB symptoms.

## Methods

### Study design and population

We conducted a prospective study of people with DM seeking care at a DM clinic at Byramjee-Jeejeebhoy Medical College-Sassoon General Hospitals (BJMC-SGH) in Pune, Maharashtra. BJMC-SGH is a large public sector tertiary care teaching institution that serves approximately 7 million population in the surrounding urban, semi-urban and rural populations. The DM clinic has 1500 adults with DM registered for care and visit the clinic monthly.

### Study procedures

Adults (age ≥ 18 years) with known DM were consented to participate. Enrolled participants were administered a questionnaire to collect demographics and medical history, including TB history and TB risk factors. All enrolled participants underwent anthropometric assessments, a baseline random blood sugar test and HbA1c test. A trained research staff administered the WHO symptom screen questionnaire that included screening for cough for longer than 2 weeks, fever, weight loss, loss of appetite, or presence of enlarged lymph glands [1]. Any positive symptoms triggered sputum smear microscopy, gene Xpert and TB culture investigations. Spontaneously expectorated sputum was collected on two occasions. All sputum specimens collected underwent digestion and decontamination by using NaOH-NALC method after direct AFB staining, and Xpert® MTB/RIF assays were performed. After centrifugation, the sediment was placed on smears for AFB staining and approximately 10 μl was inoculated on both Mycobacterial Growth Indicator Tube (MGIT liquid culture) and Lowenstein-Jensen (LJ) slant before incubation. The slants were observed for growth. People with DM were screened using symtpm screen for TB at each visit to the DM clinic over a 12 month period. The Institutional Review Board of Johns Hopkins University and the ethics committee at BJMC-SGH approved the project.

### Statistical analysis

Data were analyzed using STATA (version 13.1). Assuming 0.5% new TB cases could be detected by symptom screen based on a prior study from India [14], we estimated to detect 4–16 TB cases among 1500 DM patients. The primary outcome of the study was prevalent TB. Secondary outcomes were frequency of TB risk factors among poorly controlled DM (defined as HbA1c ≥7%) and controlled DM (HbA1c <7%). Baseline and time-updated categorical and continuous variables are summarized using proportions and medians with interquartile range (IQR), respectively, and compared using chi square test, Fisher’s exact test or Wilcoxon rank-sum test as appropriate between adults with controlled DM and poorly controlled DM; *P*-values less than 0.05 were deemed statistically significant.

## Results

### Study population characteristics

Of 675 adults with DM who visited the clinic during the study period, 630 consented for TB screening; 350 (56%) were female, and the median age was 60 years (IQR: 50–65). Median HbA1c was 7.6 (IQR: 6.4–9.3) and 444 (70.5%) were defined as poorly controlled DM. Overall, 32% had a lower income at Rs. ≤10,000 and 32% were overweight (body mass index, BMI >25 kg/m2). As shown in the Table 1, the demographic and clinical characteristics between controlled and poorly controlled DM are comparable. The majority (97%) of people with DM were on oral hypoglycemics, however, compared to those with controlled DM, those with poorly controlled DM were more likelly to be on sulfonylurea (55% vs. 38% vs. *P* = 0.0001) and metformin (82% vs. 75%, *p* = 0.02).

**Table 1:** Demographic and clinical characteristics and risk factors for tuberculosis among people with diabetes mellitus (DM) by glycemic status

	Overall (*n* = 630)	Controlled DM (*n* = 186)	Poorly controlled DM (*n* = 444)	*P value*
Demographic characteristics, n (%)				
Female sex	350 (56%)	100 (54%)	250 (56%)	0.5037
Median age, y (IQR)	60 (50–65)	61 (54–66)	60 (50–65)	0.00
Marital status				0.013
Other	125 (20%)	33 (18%)	92 (21%)	
Married	493 (78%)	145 (78%)	348 (78%)	
Unmaried	12 (2%)	8 (4%)	4 (1%)	
Employed	467 (74%)	134 (72%)	333 (75%)	0.44
Monthly household income				0.87
<5000	182 (29%)	125 (28%)	57 (31%)	
(5000–10,000)	201 (32%)	142 (32%)	59 (32%)	
>10,000	132 (21%)	94 (21%)	38 (20%)	
Diabetes Mellitus characteristics, n (%)				
Median duration of DM, years (IQR)	6 (3–10)	6 (3–11)	4 (2–7)	0.00
Median HbA1c, (IQR)	7.6 (6.4–9.3)	6.5 (6.45–6.65)	8.5 (7.5–9.85)	0.00
Oral hypoglycemics				
Sulfonylurea	314 (50%)	71 (38%)	243 (55%)	0.00015
Metformin	506 (80%)	140 (75%)	366 (82%)	0.02
Injectable insulin	21 (3%)	5 (3%)	16 (4%)	0.56
Tuberculosis risk factors, n (%)				
Past history of TB	44 (7%)	17 (9%)	27 (6%)	0.16
Recent contact with TB case	13 (2%)	7 (4%)	6 (1%)	0.07
Tobacco smoking	24 (4%)	9 (5%)	15 (3%)	0.38
Alchohol abuse	30 (5%)	8 (4%)	22 (5%)	0.69
Body mass index (kg/m^2^)				0.11
Underweight (<18.5)	8 (1%)	4 (2%)	4 (1%)	
Normal (18.5–24.9)	150 (24%)	34 (18%)	116 (26%)	
Overweight (25–29.9)	200 (32%)	60 (32%)	140 (32%)	
Tuberculosis symptoms, n (%)				
Overall Cough	66 (11%)	19 (10%)	47 (11%)	
Cough >2 weeks	19 (3%)	8 (4%)	11 (3%)	0.24
Fevers	36 (6%)	9 (5%)	27 (6%)	0.54
Night sweats	54 (9%)	14 (8%)	40 (9%)	0.52
Weight loss	59 (9%)	18 (10%)	41 (9%)	0.97
Any of the four symptoms	111 (18)%	35 (19%)	76 (17%)	

### TB prevalence and risk factors

Eigteen percent of participants reported any TB symptoms; 3% reported cough, 6% fever, and 9% weight loss and 9% night sweats (Table 1). However, no prevalent TB cases were identified. Forty-four (7%) reported a prior TB history; of these, 5 (11%) reported prior TB within two years of DM diagnosis, 22 (50%) had a TB diagnosis prior to DM diagnosis and 14 (32%) had the diagnosis three years after DM diagnosis. Only 13 (2%) reported a recent contact with a known case of TB. Additional known TB risk factors, smoking (4%), alcohol abuse (5%) lower body mass index (1%) were not common and were not statistically different between people with controlled DM and poorly controlled DM.

## Discussion

In our study, the WHO-recommended active TB case finding strategy using a standardized symptom screen did not yield any new prevalent TB cases among patients with DM, even when over two-thirds had poorly controlled DM. Similar findings have been reported from South Africa and Guinea Basseau [15, 16]. In contrast, a prior cross sectional study in India that used a TB symptom-screen approach within routine health services among people with DM detected high rates of prevalent TB (600–950 cases per 100,000) but a large proportion of those newly detected were previously diagnosed and were on treatment prior to screening [14]; only 0.5% were newly diagnosed TB, confirming that TB symptom screen yield may be very low for detecting new TB among those with DM. While the joint WHO and IJTLD framework’s and Bali declaration’s recommendation of screening for active TB among people with DM is an important strategy, the feasibility of scaling up a very low yield strategy may not be attractive to national programs. Furthermore, cost-effectiveness of such a strategy is unknown and emerging evidence suggests that symptom screening may poorly predict TB disease in high risk groups [17, 18]. Whether enhancing the screening strategy using Xpert® MTB/RIF improves yield is yet to be studied.

Whether TB patients with diabetes are less symptomatic has not been supported by prior research that showed more severe or similar clinical presentation of TB among patients with DM [19, 20]. We found that TB risk factors among people with DM in our setting were fairly low as compared to the general population in India [21]. One interesting finding is that 7% of the people with DM reported prior TB history, and over 50% of prior TB was diagnosed before a DM diagnosis. Of these, 11% reported previous TB diagnosis within the first 2 years of DM diagnosis [22]. Screening for TB among people with DM is now strongly recommended in India [6, 23], though there is a need to identify specific factors among people with DM that increase risk of TB so that screening can be targeted and effective. Understanding the basic epidemiology including incidence and prevalence of TB infection and disease and timing and rate of acquisition or reactivation of TB disease among people with DM is urgently needed to define control strategies.

Our study has several limitations. We only conducted microbiologic investigations on people with symptoms as this is the current recommendation. Secondly, while we included all people with DM consenting to be screened for TB longitudinally, our sample size was small and was restricted to one site. Although our initial estimate was that 4–16 TB cases could be detected among 1500 DM patients, we enrolled only 630 participants. However, even with this sample size, we expected to see 3–16 cases of TB. We did not collect additional information such as lipids, kidney function or blood pressure. Although we did not exclude type 1 DM, the majority of patients who visited our DM clinic were type 2 DM patients. Finally, we did not perform radiologic investigations among those with positive TB symptoms, potentially underestimating the TB prevalence. Nonetheless, our study addresses an important question on whether the existing recommendation of TB screening strategy will have utility for India’s program where convergence of these two epidemics burdens the health system disproportionately. Furthermore, our study provide a phenotype of DM in this seting.

## Conclusions

Our study demonstrated that a TB screening strategy using the WHO-recommended symptom screen questionnaire may be inadequate among people with DM in India. High TB risk populations among people with DM must be identified if TB screening is to be feasible in settings such as India where the DM epidemic continues to rise. Future studies should confirm our findings; distinguish people with DM at greatest risk for TB and most feasible diagnostic algorithms for detecting TB among this population; and assess whether targeting persons with DM who are close contacts of TB or who have established risk factors such as longer duration of DM, poor glycemic control, heavy smokers, alcohol users and lower body mass index would be appropriate strategies, as these factors may independently increase TB risk.

## Additional file

Additional file 1: Supplementary file: Raw data of the TB screening in patients with diabetes study. (XLSX 497 kb)

## Abbreviations

AFB: Acid fast bacilli
DM: Diabetes mellitus
HIV: Human immunodeficiency virus
IUATLD: International Union against tuberculosis and lung disease
TB: Tuberculosis
WHO: World Health Organization
